# Single-Institution Experience with Selective Internal Radiation Therapy (SIRT) for the Treatment of Primary and Secondary Hepatic Tumors

**DOI:** 10.7759/cureus.7628

**Published:** 2020-04-10

**Authors:** Kabalan Yammine, Francois Kamar, Jason Nasser, Claude Tayar, Marwan Ghosn, Feras Chehade, Jihad Daher, Gregory Nicolas

**Affiliations:** 1 Radiology, Clemenceau Medical Center, Beirut, LBN; 2 Oncology, Clemenceau Medical Center, Beirut, LBN; 3 Medicine, Lebanese American University School of Medicine, Beirut, LBN; 4 Surgery, Clemenceau Medical Center, Beirut, LBN; 5 Hematology and Oncology, Clemenceau Medical Center, Beirut, LBN; 6 Hematology and Oncology, Faculty of Medicine, Saint Joseph University, Beirut, LBN; 7 Nuclear Medicine, Clemenceau Medical Center, Beirut, LBN; 8 General Surgery, Lebanese American University Medical Center, Beirut, LBN

**Keywords:** vascular and interventional radiology, interventional oncology, radioembolization, liver tumors

## Abstract

Purpose: We aim to provide results of the real-world experience of a single center in Lebanon on the use of radioembolization to treat liver-only or liver-dominant tumors.

Methods: This retrospective review included patients who were evaluated for radioembolization between January 2015 and June 2017 and who had a lung shunt fraction of 20% or less. Tumor responses were determined using the response evaluation criteria in solid tumors (RECIST).

Results: Of the 23 Arab patients with a median age of 64 years (range, 36-87 years), eight had hepatocellular carcinoma, four had cholangiocarcinoma, and 11 had liver-only or liver-dominant metastases from other primary cancers. Most (n=17) had multifocal lesions, and 13 had a history of branched (n=8) or main (n=5) portal vein thrombosis. When appropriate, the gastroduodenal artery and middle hepatic artery were embolized for consolidation of radiotherapy; 18 patients required arterial coil occlusion, two had their cystic artery occluded, and one developed cholecystitis, which was successfully treated with antibiotics and supportive care. Another patient developed a post-radioembolization complication-a peptic ulcer unrelated to arterial reflux of microspheres because both the gastroduodenal and right gastric arteries were occluded. The median time to progression was seven months (range, 3-36 months), and median overall survival from radioembolization was 12 months (range, 3-40 months). Tumor responses included five complete responses, 13 partial responses, one stable disease, and four cases of progressive disease.

Conclusion: Performing radioembolization in a non-referral, private center in Lebanon resulted in good patient outcomes with few complications.

## Introduction

Even though a paucity of cancer research in the Middle East exists, the incidence of cancer in the region, especially in Lebanon, continues to rise [1,2]. Primary liver malignancies are common, being the fifth and ninth most frequently diagnosed malignancy in adult men and women, respectively [3]. Secondary or metastatic liver malignancies are even more common and prove to be a significant cause of morbidity and mortality [3-5]. 

Transarterial radioembolization involves the image-guided percutaneous injection of yttrium-90 (Y-90) microspheres directly into the arteries that feed liver tumors. This application of brachytherapy is particularly valuable in the treatment of primary and secondary liver tumors, considering the radiosensitivity of the liver and its limiting intolerance to external beam radiotherapy [6]. The consensus is that sufficient evidence supports the safety and efficacy of Y-90 microsphere therapy in the treatment of hepatic tumors, both primary and secondary [6,7].

Radioembolization is particularly efficient as it capitalizes on certain physiological phenomena. Approximately 80% of the blood supply to the normal liver tissue stems from the portal vein, while 90% to 100% of the blood supply to liver tumors stems from the hepatic artery [8,9]. Furthermore, tumors often have a rich blood supply, as angiogenesis is a critical factor in tumorigenesis [10]. The extensive vascularization of tumors and the relatively narrow diameter of the distal capillary beds allow the selective accumulation of microspheres in the tumor’s microvasculature. Furthermore, the differential blood supply and advantageously narrow range of Y-90 emission provide effective delivery of high doses of radiation to tumor cells while sparing normal parenchyma [9]. The tumoricidal potential of radioembolization is largely attributable to the focal high-energy beta-radiation emission of Y-90, leading to double-stranded DNA breakage and cell death, with the embolic aspect contributing little to its effect [9,11]. 

For the treatment of unresectable hepatocellular carcinoma (HCC), the safety and efficacy of Y-90 microsphere therapy have been well established in large cohort studies, with median overall survival (OS) after radioembolization ranging from 8 to 14.4 months [12,13]. When combined with systemic chemotherapy, radioembolization in patients with liver-only or liver-dominant metastases affects disease control within the liver and may extend survival; however, hepatic tolerance to radioembolization may influence patient selection for the combination treatment [14,15]. 

This study aimed to retrospectively examine the survival of 23 patients who underwent radioembolization with Y-90 microspheres in primary and secondary hepatic tumors at a low-volume single center in Lebanon. The contribution of pre-treatment coil embolization of non-target arteries to the success and safety profile of the treatment was also evaluated.

## Materials and methods

Patients and evaluation

Approval from the institutional review board committee was obtained for this retrospective review, and patient data were de-identified. Patients who were seen in the interventional radiology department for the treatment of primary or secondary liver tumors between January 2015 and June 2017 at Mount Lebanon Hospital, Beirut, Lebanon, were included. Patients were included if they had unresectable primary HCC or metastatic liver disease refractory to chemotherapy. Patients with calculated lung shunt fractions greater than 20% were excluded from radioembolization and, therefore, not included in this review.

Patients’ baseline liver function was assessed before radioembolization. Eastern cooperative oncology group (ECOG) performance status, Barcelona clinical liver cancer (BCLC) staging, and Child-Pugh class were determined for patients with HCC. 

Radioembolization protocol

Imaging by computed tomography (CT) or magnetic resonance and 18F-fluorodeoxyglucose (FDG) positron emission tomography (PET)/CT was obtained by the treating physician to establish a baseline and evaluate the proportion of tumor in the liver and the extent of extrahepatic disease. In addition, a pre-treatment angiogram was performed to evaluate the anatomy of the vascular tree and identify branches of the hepatic artery for appropriate coiling. The gastroduodenal artery (GDA) was coiled if its origin was within 2 cm of the hepatic bifurcation; the right gastric artery (RGA) was coiled if its origin was distal or close to the position of the infusing microcatheter, and the middle hepatic artery (MHA) was coiled if it was found to be contributing to the tumor supply. Coiling of the cystic artery was performed if it was found close to the infusion site and contributing to the tumor supply. 

**Figure 1:**
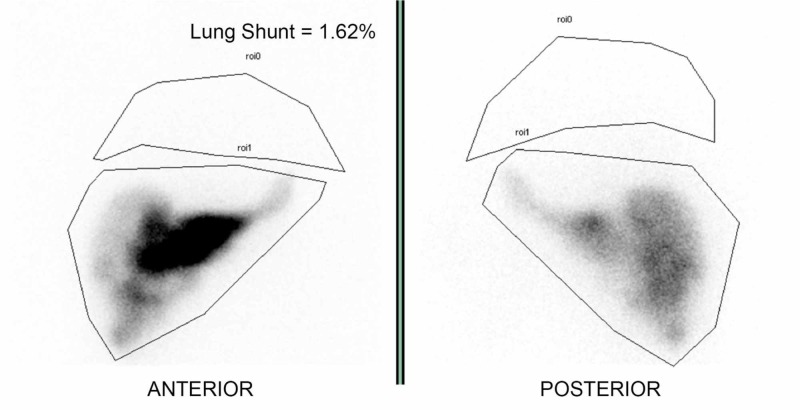
99mTc-labeled macroaggregated albumin scintigraphy 99mTc-labeled macroaggregated albumin scintigraphy is the only method available to calculate the fraction of arterially injected particles reaching the lungs. Calculations used are based on the geometric mean obtained with anterior and posterior conjugated views using regions of interest (ROIs) for liver and lung activities.

After the anatomical mapping of the hepatic arterial tree and coiling of the appropriate arteries, an attempt was made to detect hepatic-extrahepatic vascular shunts, particularly assessing shunts between the hepatic arterial system to that of the lungs or the gastrointestinal tract, by administering 4 mCi of 99mTc-labeled macroaggregated albumin through a catheter. Subsequently, gamma imaging was obtained, and regions of interest were drawn around the liver and lungs in anterior planar images (Figure 1). Images were evaluated for extrahepatic uptake, and the pulmonary shunt was calculated. Significant uptake to the gastrointestinal tract guided prophylactic coil embolization of arteries, and patients with significant uptake to the lungs beyond 20% were excluded from radioembolization. Catheter tip placement in the hepatic arterial tree to obtain the values for extrahepatic shunting was identical to tip placement in the subsequent administration of Y-90 microspheres.

Doses were calculated using the body surface area model. Radioembolization was performed using Y-90 resin microspheres (SIR-Spheres®, Sirtex Medical Limited, Sydney, Australia). The Y-90 resin microspheres have a half-life of 64.1 hours, a carrier size of 20 to 60 μm in diameter, a mean and maximum emission range of 2.5 mm and 11 mm, respectively [11,16]. 

Tumor response was assessed using response evaluation criteria in solid tumors (RECIST). Survival was measured from the time of radioembolization until death or censoring in January 2018.

## Results

Of the 23 patients receiving radioembolization, 14 were men and nine were women, all were Arab with a median age of 64 years (range, 36-87 years, Table 1). Patients had an ECOG performance status score of 0 (n=7), 1 (n=14), or 2 (n=2) and bilirubin levels <2 mg/dL (median 0.7; range, 0.08-1.40 mg/dL). Patients were diagnosed with unresectable primary HCC (n=8) or cholangiocarcinoma (n=4) or liver metastases from the following primary malignancies: colorectal carcinoma (n=3), pancreatic cancer (n=3), neuroendocrine tumor (n=1), endometrial carcinoma (n=1), ovarian carcinoma (n=1), esophageal adenocarcinoma (n=1), and ocular melanoma (n=1). Etiologies of HCC included hepatitis B or C (n=3), alcoholic liver disease (n=3), and nonalcoholic steatohepatitis (n=2). BCLC stages were A (n=1) or C (n=7), and Child-Pugh class was A (n=6) or B (n=2).

**Table 1:** Patient characteristics and baseline values

Characteristic	Value
Age in years, median (range)	64 (36–87)
Male, n (%)	14 (61)
Ethnicity (n)	Arab (23)
Portal vein thrombosis, n	
Branched	8
Main	5
ECOG performance status, n	
0	7
1	14
2	2
BCLC Stage for HCC patients only, n	
A	1
B	0
C	7
Child-Pugh Class for HCC patients only, n	
A	6
B	2
C	0
Tumor, n	
HCC	8
CC	4
Metastatic colorectal carcinoma	3
Metastatic pancreatic cancer	3
Metastatic endometrial cancer	1
Metastatic esophageal adenocarcinoma	1
Metastatic neuroendocrine tumors	1
Metastatic ocular melanoma	1
Metastatic ovarian cancer	1
Bilirubin, median (range)	0.7 mg/dL (0.08–1.40 mg/dL)
Tumor Volume, n (%)	
<24%	13 (57%)
25-49% 50-70%	6 (26%)
50-70%	4 (17%)
Number of lesions, n (%)	
1	5 (22%)
2	1 (4%)
Multifocal lesions	17 (74%)
Bilobar disease, n (%)	16 (70%)
Pre-treatment arterial coil occlusion, n	
Gastroduodenal artery	14
Right gastric artery	13
Middle hepatic artery	4
Cystic artery	2

All patients had lung shunts <20% (median 5.0%; range, 1%-19%). Initial tumor volume was <24% in 13 patients (57%), 25% to 49% in six patients (26%), and 50% to 70% in four patients (17%). Five patients (22%) had a single lesion, one patient (4%) had two lesions, and 17 (74%) had multifocal lesions. Seven patients (30%) received unilobar treatment, while 16 (70%) received bilobar treatment in a single session. Bilobar treatment was chosen to prevent delay in treatment and risk of tumor progression in an untreated lobe, as well as to avoid the added prohibitive cost of splitting the treatment into two sessions over weeks to months. Eight patients (35%) had a history of branch or main portal vein thrombosis. 

**Figure 2:**
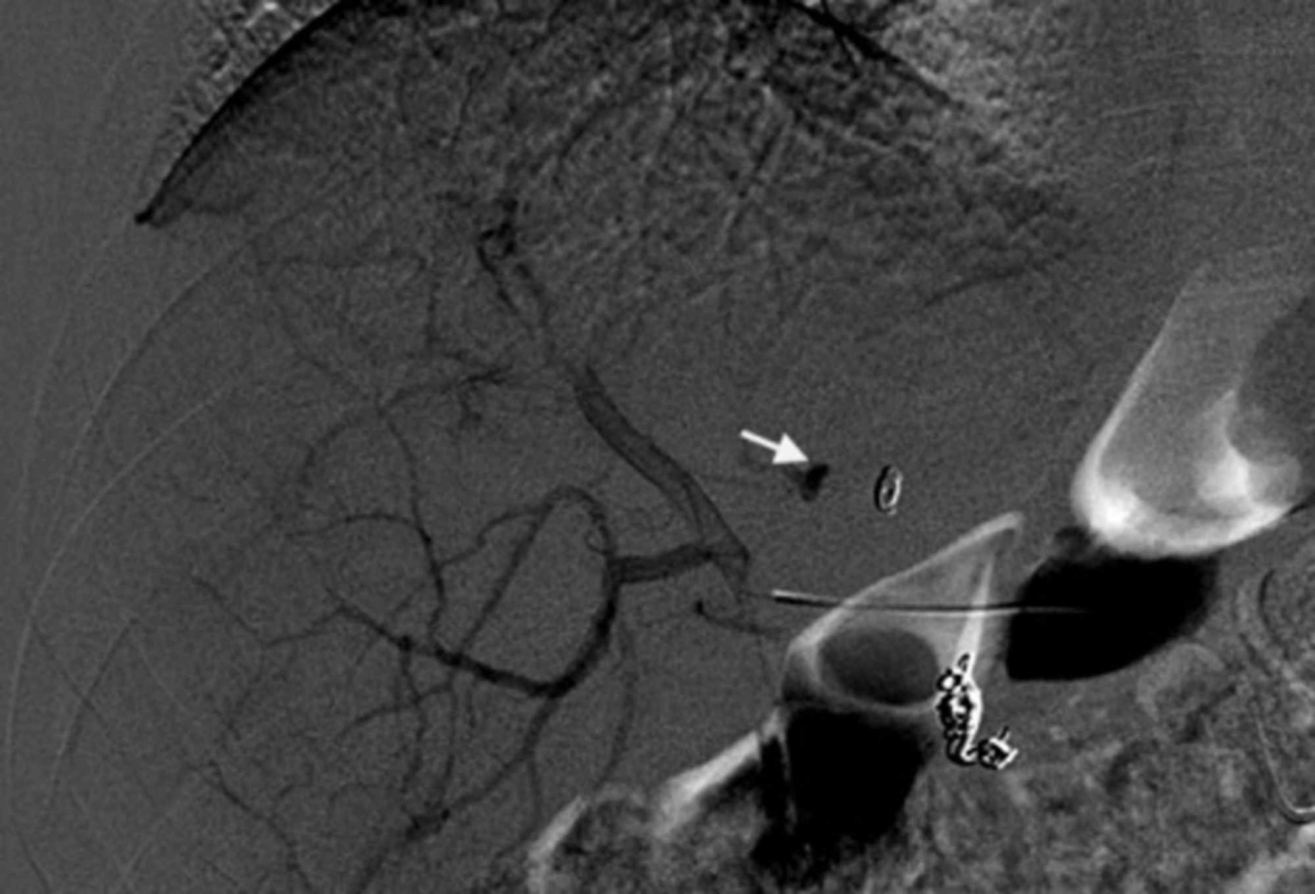
Preparatory angiogram of patient with rectal cancer and liver metastasis before radioembolization The aortogram shows the middle hepatic artery (arrow) with a coiled gastroduodenal artery.

**Figure 3:**
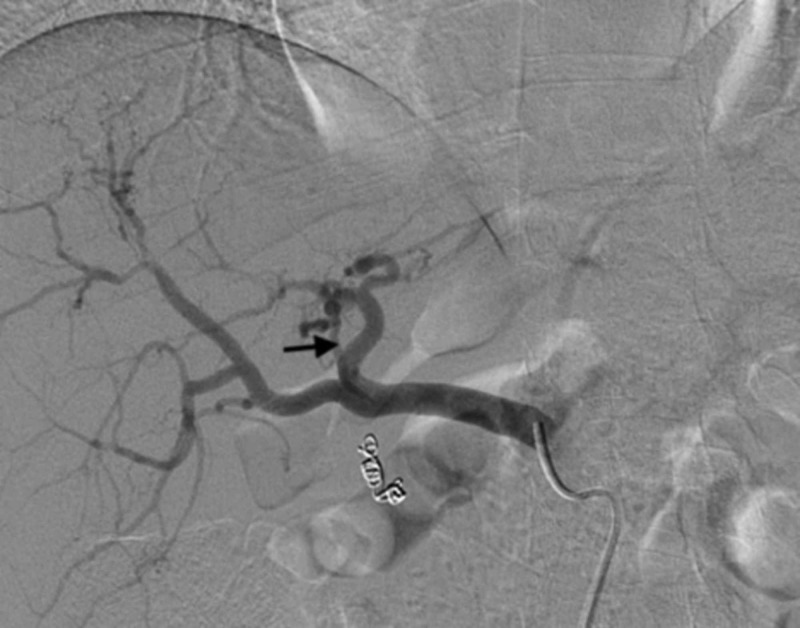
Status post coiling of the middle hepatic artery (arrow) for redistribution and consolidation of the hepatic arterial flow

Of the 23 patients, 18 required a coil occlusion of one or more arteries: the GDA was coiled in 14 patients, the RGA in 13 patients, and the MHA in four patients. Figures 2, 3 show the successful coiling of the GDA and MHA, respectively. The cystic artery was coiled in two patients. The falciform ligament was not coiled in any patient, and no skin ulcers were observed. 

**Figure 4:**
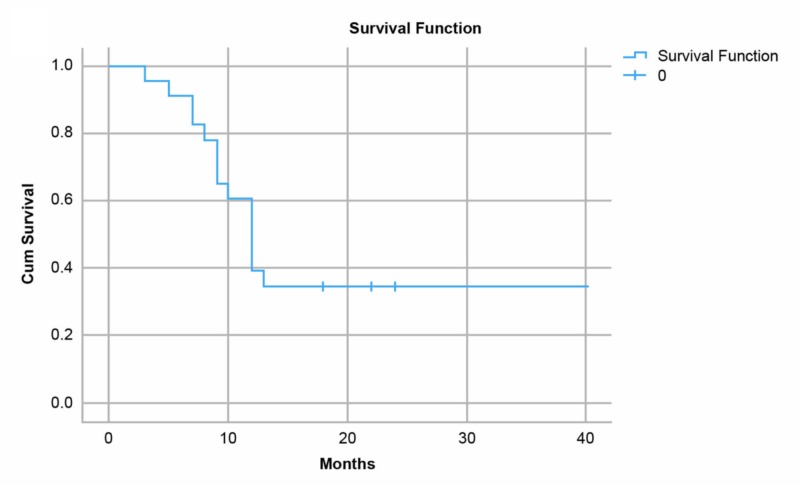
Kaplan-Meier curves for patients after undergoing transarterial radioembolization with resin microspheres for hepatocellular carcinoma or liver metastases showing overall survival

**Figure 5:**
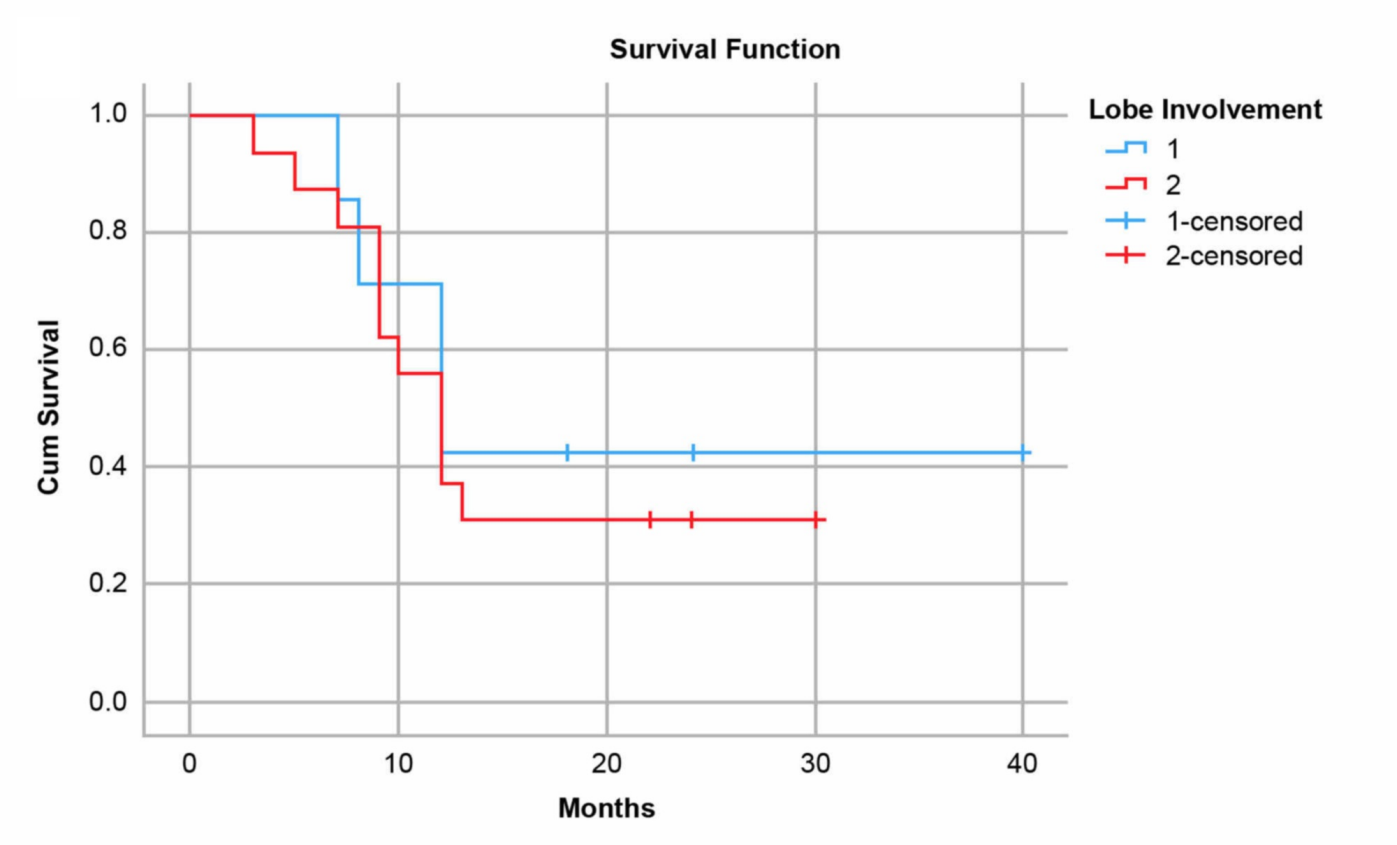
Kaplan-Meier curves for patients after undergoing transarterial radioembolization with resin microspheres for hepatocellular carcinoma or liver metastases showing survival by lobe involvement

**Figure 6:**
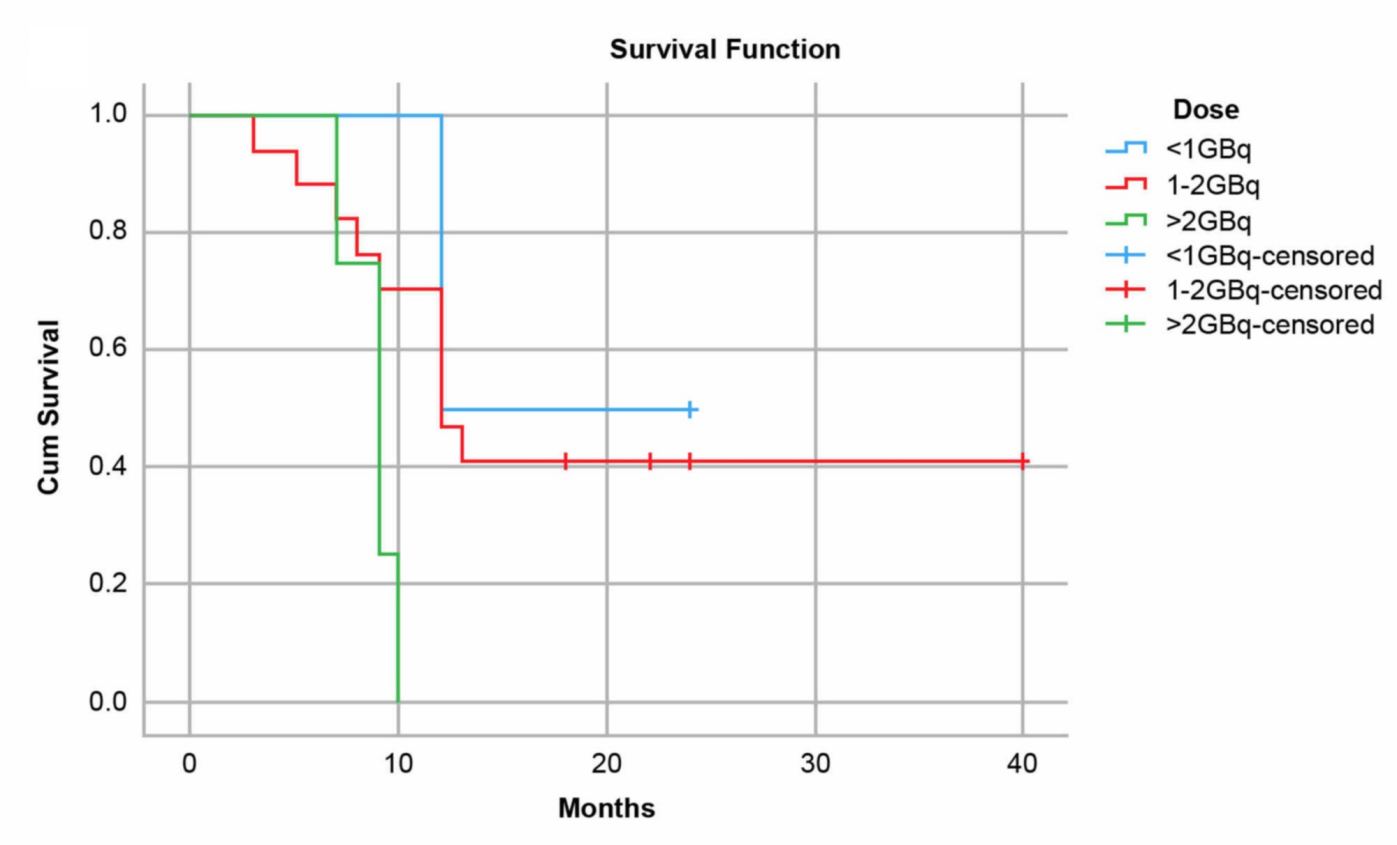
Kaplan-Meier curves for patients after undergoing transarterial radioembolization with resin microspheres for hepatocellular carcinoma or liver metastases showing survival by dose

**Figure 7:**
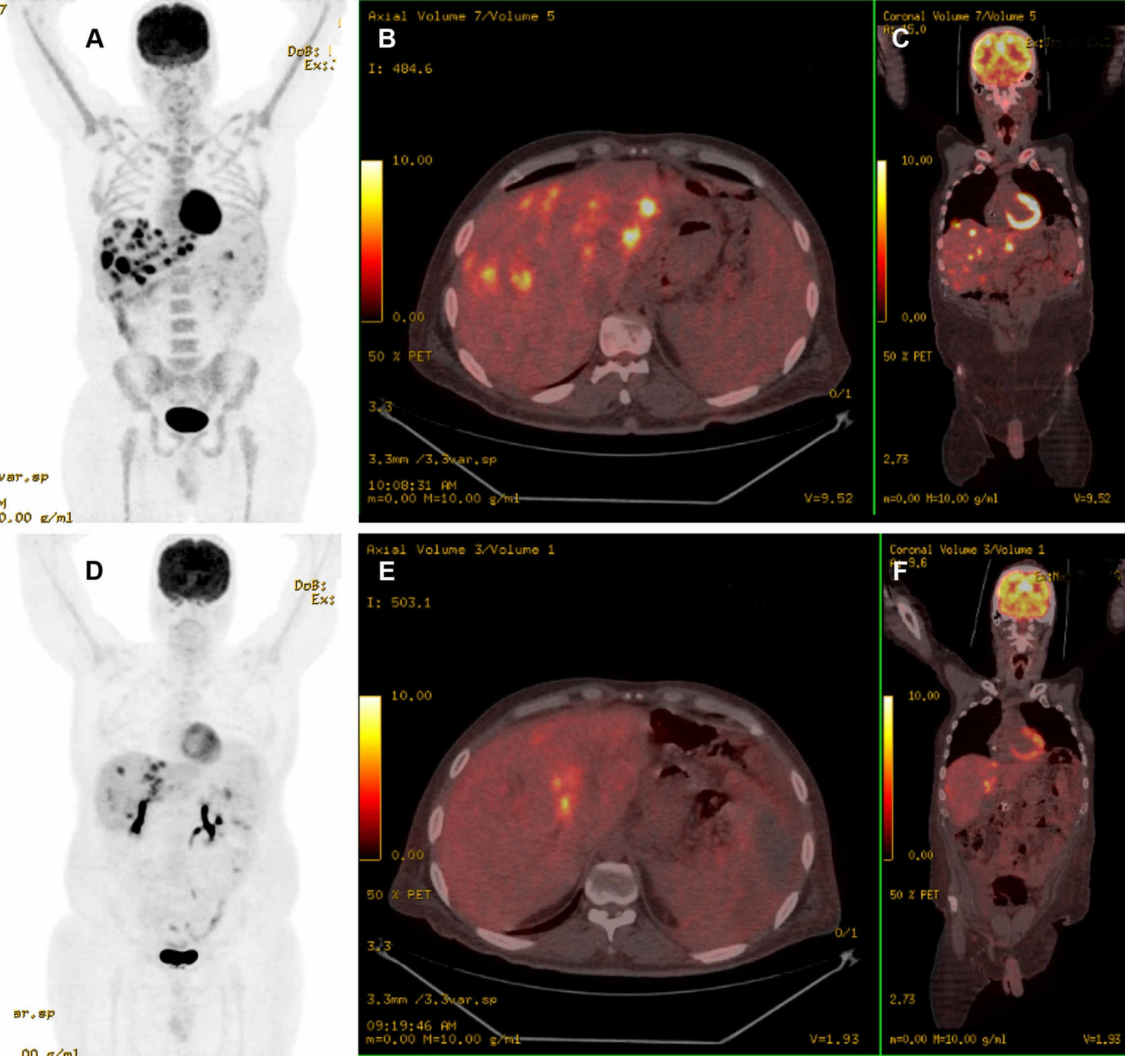
18F-fluorodeoxyglucose positron emission tomography–computed tomography (18F-FDG PET/CT) staging of pancreatic neoplasm in 38-year-old man Innumerable hypermetabolic tumor implants were scattered in the left and right lobes of the liver as shown in the 3D image (A) and on the transverse (B) and coronal (C) views. Four months after radioembolization with Y-90 microspheres, 18F-FDG PET/CT imaging shows a partial therapeutic response of the liver tumors as demonstrated on the 3D image (D) and on the transverse (E) and coronal (F) views.

**Figure 8:**
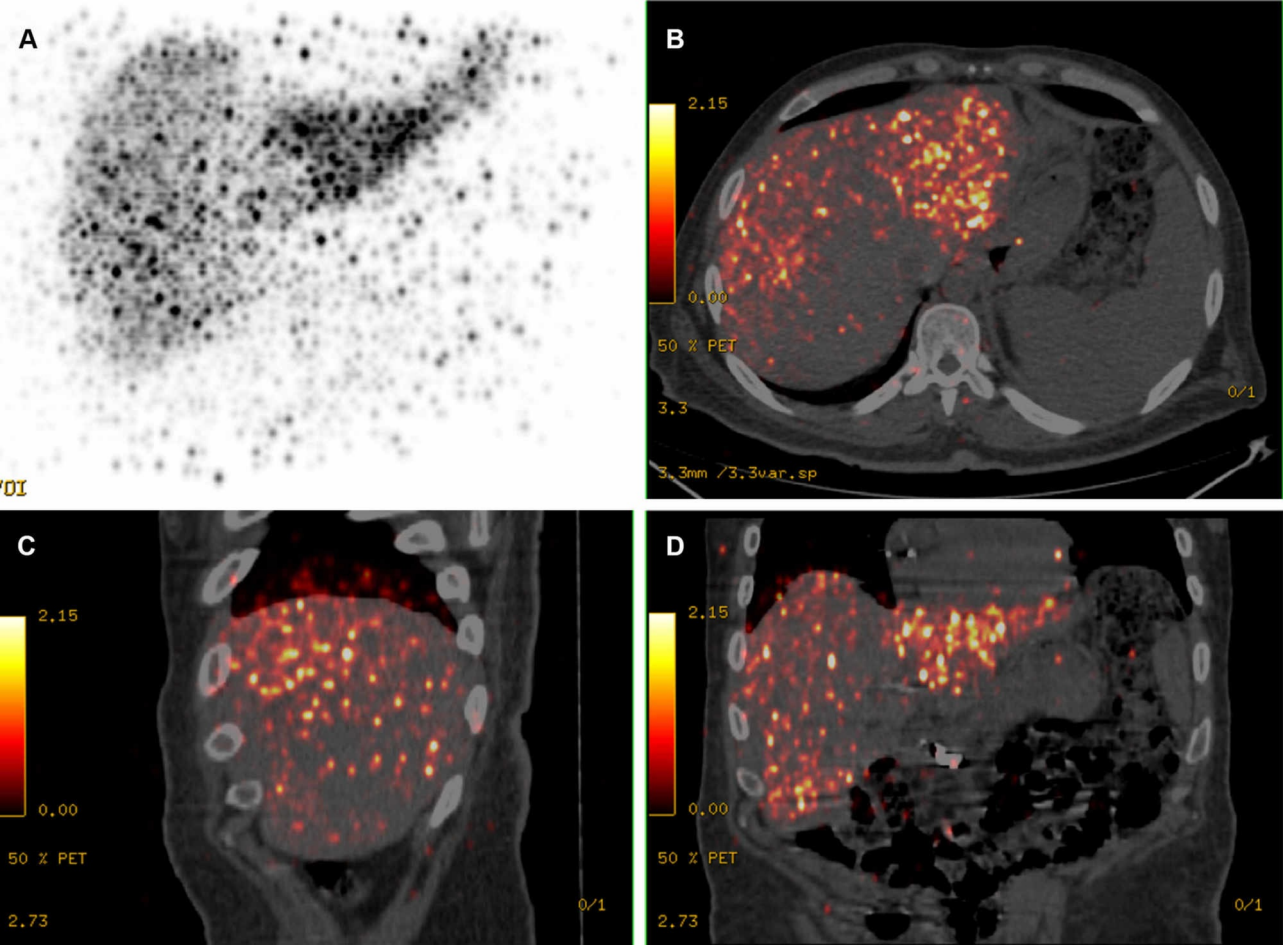
Y-90 positron emission tomography–computed tomography (PET/CT) imaging Following radioembolization, Y-90 positron emission tomography–computed tomography (PET/CT) imaging shows non-homogeneous distribution of Y-90 microspheres inside the hepatic parenchyma, as revealed on the 3D PET (A) image and the PET/CT fusion transverse (B), sagittal (C), and coronal (D) views.

The median time to progression was seven months (range, 3-36 months), and median OS from radioembolization was 12 months (range, 3-40 months; Figure 4). Survival curves based on lobar involvement and dose are provided (Figures 5, 6). Tumor responses included a complete response for five patients and partial response for 13 patients; one patient had stable disease, and four had progressive disease. Representative images showing microsphere distribution and responses are provided (Figures 7, 8).

Only two patients had postoperative complications. One patient had a peptic ulcer. This patient had undergone coiling of both the RGA and GDA, but the ulcer was not due to arterial reflux of microspheres. Biopsy by esophagogastroduodenoscopy of the gastric ulcer revealed both the presence of tumor invasion and Y-90 microspheres, which indicated that the peptic ulcer was due to direct tumor invasion of the stomach. This invasion was not detected on pre-operative CT of the abdomen. The other patient developed cholecystitis, most likely secondary to the coiling of the cystic artery. Coiling was necessary for the consolidation of arterial flow, as tumor feeders from the cystic artery were present. This patient also had multiple stones in the gallbladder; the presence of stones in the gallbladder is known to lead to baseline intramural vascular insufficiency [17]. The cholecystitis was successfully treated with antibiotics and supportive care and did not require cholecystectomy. Only one other patient underwent cystic artery coiling but did not experience any complications.

## Discussion

This study is the first to report on the real-world experience of treating patients with HCC or liver metastases using radioembolization with resin microspheres in the Middle East. It demonstrates the feasibility of performing transarterial radioembolization in the treatment of both primary and secondary liver tumors in a low-volume, non-referral, private medical center with subsequent survival benefit and a low rate of complications. Median OS for patients included in our study is comparable to other recent studies involving mixed tumors types, ranging from 11.7 to 19.0 months [18,19]. 

Radioembolization is an effective treatment for both primary and secondary hepatic tumors; however, it is an intricate procedure that necessitates an intelligent approach in its application. A core step in the procedure is the pre-treatment angiogram that maps the arterial tree and identifies hepatico-enteric communicating vessels that are candidates for coil embolization for endovascular skeletonization of the arterial system [7,20]. Hepatic artery anatomy, and subsequently hepatic tumor blood supply, can often be complicated, and tumors may receive blood from more than one artery, each of which would then require individual radioembolization. However, an alternative exists for this cumbersome procedure. Consolidation of arterial flow, as done by the coiling of the MHA for our patients, is a technique that leads to vascular flow redistribution through intrahepatic collateral pathways [21-23]. Ischemic hepatic injury resulting from the coiling of variant arterial anatomy is rare, and as such, concern is minimal [22-26]. Coiling allows for better delineation and isolation of tumor blood supply and offers a more targeted and complete treatment that better spares healthy liver parenchyma. This approach also simplifies the procedure by reducing the number of administration sites and the amount of Y-90 microspheres required to achieve the same dose of radiation. 

The decision to coil was based on multiple factors. MHA coiling was avoided in patients whose tumors were not perfused by the MHA, as there would be no therapeutic advantage in that case. Coiling of the GDA and RGA was done if the artery was near or distal to the infusion site to avoid non-target radioembolization and ulceration as a result of the reflux of Y-90 microspheres to the stomach, pancreas, or small intestine [27]. Universal coiling of the GDA was not performed, and this did not affect the treatment’s safety profile, which is similar to other reports [25,28].

Of the two patients who experienced serious adverse events, one required coiling of the cystic artery. Coil embolization of the cystic artery is a complex decision because it requires balancing the risk of radiation cholecystitis secondary to non-target radioembolization against the risk of ischemic cholecystitis secondary to coiling of the cystic artery. This measure is, therefore, only considered if it is impossible to otherwise avoid infusing the cystic artery. It is inaccurate to consider the gallbladder as an end-artery organ as it receives collaterals from the hepatic gallbladder bed and the GDA, and collaterals often play a more significant role than the cystic artery, making safe coil embolization more likely [29,30]. Recent studies indicate a lack of benefit to prophylactic coiling of the cystic artery [30]. However, some indicators that should inform the decision to coil include the origin of the cystic artery and the choice of microspheres [29]. Nevertheless, specific criteria have yet to be identified that accurately predict who may be most at risk for either ischemic or radiation cholecystitis [29].

Our study has several limitations, including its retrospective and non-randomized nature. The patient population was significantly heterogeneous, with both primary HCC and various secondary tumors, many of which carry a worse prognosis, such as pancreatic adenocarcinoma. Furthermore, the small population size allowed for limited analysis of survival and precluded stratification by tumor origin. 

## Conclusions

This study demonstrates the feasibility of performing transarterial radioembolization in the treatment of both primary and secondary liver tumors in a low-volume, non-referral, private center in Lebanon, with good disease response and a low rate of complications. Furthermore, a positive experience with the coiling of the GDA and MHA for consolidation of radiotherapy when appropriate, while avoiding prophylactic coiling, is reported, consistent with the literature. However, cystic artery coil embolization appears to carry a high risk of sequelae, particularly if the patient already has a diseased gallbladder. 
